# Deepfake defense: Combining spatial and temporal cues with CNN–BiLSTM–transformer architecture

**DOI:** 10.1371/journal.pone.0334980

**Published:** 2025-11-14

**Authors:** Srijana Yadav, S. Sudheer Mangalampalli

**Affiliations:** Manipal Institute of Technology Bengaluru, Manipal Academy of Higher Education, Manipal, India; University of Sargodha, PAKISTAN

## Abstract

The proliferation of deepfakes is a major threat to the believability of online media and the stability of public discourse. These hyper-realistic fake videos, nearly indistinguishable from genuine content, can be misused to spread disinformation, conduct identity theft, and manipulate political narratives. Most existing deepfake detectors analyze spatial or temporal features in isolation however, in real-world scenarios involving video compression, occlusions, or frame instability, such approaches are inadequate. Convolution Neural Networks (CNN) effectively capture spatial artifacts but fail to model temporal dynamics, while recurrent neural networks (RNNs) and long short-term memory (LSTM) units handle short-range temporal signals but struggle with long-term dependencies. To address these limitations, we propose a hybrid deep learning architecture that integrates CNN, bidirectional LSTMs (BiLSTMs), and transformer encoders within a unified framework. The CNN module extracts fine-grained spatial information from each frame, the BiLSTM branch captures local temporal motion, and the transformer encoder models global temporal relationships across video sequences. This dual-path temporal modeling framework leverages the strengths of both sequential learning and attention mechanisms to enable comprehensive spatiotemporal analysis. The model is implemented in TensorFlow using MobileNetV2 as its CNN backbone and evaluated on the FaceForensics++ and DeepFake Detection Challenge (DFDC) datasets. The proposed architecture demonstrates superior performance compared to baseline models such as XceptionNet, CNN–LSTM, and CNN–Transformer, achieving an F1-score of 90.6% and an AUC of 98.5%. In addition to high detection accuracy, the model exhibits strong robustness against video quality degradation, making it a practical and scalable solution for detecting deepfakes in critical and sensitive applications.

## 1 Introduction

Deepfake technology, one of the advances of contemporary artificial intelligence, facilitates the creation of hyperrealistic but completely fake multimedia materials. These manipulated videos can appear so real that telling them apart from genuine material is challenging. By taking advantage of deep learning algorithms such as GANs, deepfakes can simulate the replacement of one person’s face, voice, or entire expressions with another. The outcomes of such possibilities are huge and far-reaching, especially considering the growing popularity of deepfake-generation technology among the public. Deepfakes can be used for malicious purposes to cause great harm on various levels. On a personal level, deepfakes can be used for identity theft, extortion, or personal defamation. On a broader social level, they can undermine democratic processes, incite violence based on misinformation, and damage public trust in digital media [1,2]. Such fraudulent videos are usually impossible to distinguish with the naked eye, and identification and verification pose a significant technical and ethical problem. As their visual authenticity increases over time, traditional methods of media verification are rapidly becoming obsolete. The rapid circulation of online sharing platforms such as YouTube, TikTok, and Twitter allows these manipulations to reach millions of people with false stories within minutes [3,4]. Against this threat, the development of powerful detection tools is an international research priority. Several methods have recently emerged to identify and mitigate deepfake content [5]. However, most of these early solutions rely on spatial analysis investigating static features from a single frame or temporal analysis considering changes from one frame to the next. While useful in controlled situations, they fail to capture the full picture of manipulated content, especially in adverse real-world conditions such as video compression, occlusion, or frame dropping. Therefore, the need for more robust, dynamic, and reliable detection approaches has never been greater.

The explosion of deepfakes has also brought about a broader cultural shift in the way video evidence is interpreted. While the adage “seeing is believing” was once unassailable, deepfakes have created a climate of skepticism. Today, even authentic videos can be questioned, and the erosion of trust in the media has significant consequences for journalism, justice, and public debate [6,7]. However, since around 2017, interest in the subject has grown not only among tech enthusiasts, but also among policymakers, media monitors, and the general public. Seeing both the technical sophistication and social urgency of the challenge, the scientific community has begun to invest in cutting-edge research to learn about the inner mechanics of deepfake algorithms and to build tools to detect and counter them [8,9]. This study stands out in a growing literature, specifically aiming to detect deepfakes in video material using a hybrid deep learning model. Traditional models capture either spatial or temporal features, while our method combines both in a unified design. CNNs extract spatial details like facial attributes, lighting errors, and subtle anomalies, but these alone cannot detect temporal manipulations. To address dynamic artifacts and frame instabilities, RNNs with LSTM units are incorporated. These are good at observing local temporal relationships but struggle with long-range context due to their sequential bottleneck and shallow memory depth. Recent advances in machine learning have put transformer architectures at center stage as strong candidates for sequence modeling. Originally introduced for natural language processing, transformers exploit self-attention to learn global dependencies in sequences putting them in a natural state to analyze long-term dependencies in videos. They are computationally efficient and effective in simulating complex patterns of an entire video clip because they can process all time steps simultaneously.

We propose a deepfake detection system that combines CNNs, LSTMs, and transformers in a single architecture. Videos are first segmented into frames, and a pre-trained CNN extracts their spatial features. These are input into two parallel streams, one that processes short-term motion and temporal flow, and a transformer encoder to detect long-term dependencies in the video sequence. The outputs from the two streams are combined and then input into a classification layer that determines whether the presented video is real or doctored. This architecture leverages the respective strengths of each of the three components CNN for spatial accuracy, LSTMs for sequential perception, and transformers for global temporal perception. The resulting system is accurate and understandable, demonstrating a comprehensive approach to detecting deepfakes in a variety of scenarios. Our motivation is the increasing realism of modern deepfakes and the limitations of existing detection techniques. To be useful, a detection system must detect not only static instability but also subtle time-dependent tampering. The combination of spatial and temporal modeling, demonstrated by our hybrid framework, is an interesting path for the future development of the field of deepfakes forensics.

### 1.1 Motivation

The serval studies have explored CNN–LSTM or CNN–transformer combinations for deepfake detection, these approaches typically operate in a sequential manner and focus on local or global temporal features in isolation. Such designs limit their ability to generalize to challenging real-world situations such as video compression, frame occlusion, or resolution degradation. In contrast, our work introduces a dual-path hybrid architecture in which BiLSTM and transformer branches process CNN-extracted spatial embeddings in parallel. This design explicitly couples short-term motion cues with long-range temporal dependencies, thereby providing a richer and more balanced spatiotemporal representation of video content. To our knowledge, this is one of the first frameworks to combine CNN, BiLSTMs, and transformers in a parallel dual-path configuration, and it demonstrates superior robustness and accuracy compared to single-technique or sequential hybrid baselines.

This study advances the state of the art in deepfake detection through the following key contributions:

- We propose a novel **dual-path hybrid framework** that integrates convolutional neural networks (CNN), bidirectional long short-term memory (BiLSTM) networks, and transformer encoders into a unified architecture. Unlike existing CNN-LSTM or CNN-Transformer approaches, our design processes local and global temporal dependencies in parallel, enabling richer spatiotemporal representation.
- The framework introduces a **parallel temporal modeling scheme**, where BiLSTM networks capture short-range motion dynamics and transformer encoders model long-range temporal dependencies. The fusion of these complementary branches strengthens the system’s ability to detect subtle and complex temporal inconsistencies in manipulated videos.
- The proposed approach demonstrates **robustness under real-world conditions**, maintaining consistent performance in the presence of video compression, frame occlusion, and resolution degradation scenarios that often degrade the effectiveness of existing methods.
- Extensive experiments on benchmark datasets such as **FaceForensics++** and the **DeepFake Detection Challenge (DFDC)** show that our model outperforms the baseline works , achieving an F1-score of 90% and an AUC of 98.5%.
- A comprehensive **ablation study** validates the complementary roles of the CNN, BiLSTM, and Transformer modules, confirming that their integration in a dual-path design significantly improves recognition accuracy compared to single-technique or sequential hybrid models.

The structure of this paper is as follows: Sect 2 reviews existing methods for deepfake detection. Sect 3 describes the proposed hybrid framework, incorporating CNNs, BiLSTMs, and transformer encoders, along with the associated preprocessing pipeline. Sect 4 presents the experimental design and analyzes the results, while Sect 5 concludes the study and outlines possible avenues for future work.

## 2 Literature review

Deepfakes technology, especially video forensics, has emphasized the importance of efficient and sound identification processes. Deepfakes, which are created using artificial intelligence a serious threat of visual content and society [10]. Deepfakes can alter content by replacing one person face to another and altering speech to a level that is almost unrecognizable to the human eye [1,6]. As this becomes easier and the technology becomes more accessible, the barriers to entry for malicious actors will decrease and there will be more opportunities [11,12]. This evolving threat environment requires a robust, multi-disciplinary solution that combines computer vision, machine learning, digital forensics, and sociological capabilities [1]. Current deepfake detection approaches present this problem largely as a binary classification task - deciding whether a given video or frame is real or not [13]. The steady progress of generative models has been matched by advances in detection algorithms. This requires continuous innovation and improvement of detection mechanisms to keep up [14]. Compounding this problem is the vast amount of video content created and uploaded daily on social media websites, where lossy compression, downsampling, and platform-proprietary preprocessing pipelines hide tell-tale indicators of manipulation [1]. Researchers are therefore investigating a range of techniques, from low-level image analysis and micro-expression tracking to cross-modal asymmetries between audio, facial motion, and speech [1,15]. In addition, there is a growing focus on defining robust evaluation criteria and benchmarking datasets to objectively evaluate models and accelerate collective progress within the research community [16].

Their ability to digitally imitate individuals without using any special features other than facial resemblance adds a new dimension of threats to personal privacy, political speech, and legal credentials [17]. From a technical perspective, deepfakes use AI techniques such as autoencoders, GANs, and encoder-decoder pipelines to generate or modify visual and audio content [18]. The content produced also ranges in complexity from simple “shallow fakes” with minor modifications to frame rate or context, to highly realistic deepfakes that can replicate precise facial movements and voice inflection. As a result, distinguishing real from tampered media is now a major computational and perceptual challenge [2].

### 2.1 Approaches to deepfake generation and identification

Deepfake generation relies robust deep learning frameworks that can manipulate audio visual data. Most of these systems use encoder-decoder architectures, or GANs, to generate content that can easily mimic human appearance and behavior [6]. Generative Adversarial Network (GAN) models have demonstrated the ability to synthesize high-resolution images and videos that closely resemble authentic material. Among these, face-swapping is the most widely used approach, in which a target individual’s face is seamlessly overlaid onto another person in the source video. This technique can convincingly reproduce subtle details such as lip synchronization, eye blinking, and fine facial expressions to enhance realism [6,19].

As the technology that powers deepfakes evolves, so too must the methods used to detect them. However, despite ongoing advances in generation techniques, detection is still generally based on the same basic principles that are effective that is, finding anomalies that provide key synthetic features of the video. These can be found in facial alignment, brightness variations, unusual eye or mouth movements, or even misalignment of the face and voice [9]. With the increasing ease of creating deepfakes and the lack of visual cues that reliably enable human identification, the proliferation of automated detection systems has become imperative [1,20]. In addition, as deepfakes become more realistic, they have a much greater potential for misuse. Their ability to attach artificial actions to real identities exposes them to situations that can damage the trust and reputation of politicians, the public, and [2,17].

Therefore, recognition methods must move beyond superficial visual inspection or shallow classifiers. Recent methods use deep learning not only for classification, but also to robustly capture spatial and temporal patterns. It uses CNN for spatial processing and RNNs or transformers to model temporal relationships in video sequences [9]. These developments in recognition architectures reflect a broader move towards multi-dimensional analysis in deepfake forensics.

### 2.2 Technical solutions for deepfake detection

Several techniques have been proposed to detect deepfakes, with varying capabilities, assumptions, and limitations. A common area of research involves examining facial microexpressions and distortions of features that are not well simulated by generative models. Inconsistencies in head posture, inconsistent blink rates, unnatural lip sync, and very small time differences between audio and facial animation, for example, can be used as indicators of manipulation. Frequency-domain analysis is another important detection category, in which artifacts or compression anomalies at high frequencies introduced during the generation process are examined for unusual patterns [20] Other approaches examine anomalies, texture anomalies, or differences in color distributions in the RGB and chroma channels of video frames [21].

A newer and more promising solution centers around deep neural networks specifically Bayesian convolutional neural networks trained on vast databases of real and synthetic videos [22].These models aim to automatically identify the subtle differences between deepfake content and genuine media. Despite their accuracy, they remain vulnerable to various challenges. For instance, many deep learning systems can be deceived by adversarial attacks [23], where minor alterations in pixel values lead to incorrect predictions. Moreover, the performance of these models is highly dependent on the diversity and quality of the training datasets, which often leads to limited generalization when faced with novel manipulation methods or unseen content domains [6,12].

In [24] author proposes that Personalized Speech-Driven 3D Facial Animation with Style Preservation offers a novel framework for generating realistic 3D avatars while maintaining individual speech characteristics. Unlike current approaches that often ignore individual and emotional speech style, TalkingStyle separates style, speech, and motion with three separate encoders. A style-conditioned transformer decoder further combines these methods to create smooth, speech-synchronized animations. The approach is trained on motion and mouth movement losses to guarantee both global realism and proper lip synchronization. FaceFormer and CodeTalker outperformed in experiments on the VOCASET, BIWI, and MEAD datasets. User studies confirm TalkingStyle’s benefits in realism, lip synchronization, and style retention. Overall, this work advances personalized avatar generation by addressing many-to-many speech-to-facial movement mapping.

In [25] authors present an LG-LDM combined with an FDM to synchronize overall facial expressions with lip motion, and employ an EVQ-VAE to capture fine-grained emotional details which clusters latent codes by emotion, allowing for fine-grained reconstruction of expressive facial geometry. The system generates animations in a two-step process: first encoding facial expressions and lip shapes into emotion-aware latent codes, and then applying a conditioned diffusion model to the audio and emotion labels to synthesize the final motion. Evaluations on datasets including MEAD, RAVDESS, VOCASET, and BIWI. User studies confirm improvements in realism and expressiveness, although performance lags slightly on neutral-expression datasets. While noting limitations such as dataset bias, reliance on emotion labels, and scalability challenges, the study concludes that LG-LDM with EVQ-VAE can significantly enhance the emotional depth, lip synchronization, and real-time usability of speech-based avatars.

Traditional graph neural networks (GNNs) often assume homophily (like nodes are connected), but in fraud detection, fraudsters intentionally create heterophilic links with regular users to disguise their behavior [26]. To address this, the authors introduce H^2^IDE, which decomposes node representations into multiple latent factors to capture different interaction patterns. The framework includes a factor-level recognition task to distinguish homophilic and heterophilic connections, as well as a relationship-aware hierarchical attention mechanism to optimally integrate neighborhood information across multiple relationship types. An independence constraint based on mutual information further enhances the decoupled learning. Experiments on four real-world datasets (YelpChi, Amazon, T-Finance, and T-Social) show that H^2^IDE baselines in metrics such as ROC-AUC, F1-macro, G-Mean, and PR-AUC. Ablation studies have confirmed that dissociation, heterophily detection, and relationship-aware aggregation are all key to its effectiveness. Overall, this work demonstrates that explicitly modeling both homophily and heterophily in a dissociative manner greatly improves fraud detection accuracy and scalability across domains.

Traditional bagging and stacking approaches face limitations bagging uses fixed weights that perform poorly on unbalanced datasets, while stacking does not include specification in the weight assignment [27]. To address this, the authors introduce funk-bagging, which combines heterogeneous classifiers with an adaptive weight distribution strategy. Experiments on battery fault detection, fall detection, and motion recognition datasets show that funk-bagging consistently improves AUC compared to single classifiers, traditional bagging, stacking, and Adaboost. The results indicate an average gain of 1.81% over the base models, with particularly strong performance on unbalanced anomaly detection tasks. Existing methods struggle to fully capture both local transitions within sessions and global dependencies across sessions [28]. To address this, the authors design HDA-GCN, which integrates hierarchical modeling and a dual attention mechanism. At the session level, a graph convolutional network models item transitions, while at the global level, item embeddings are enhanced by cross-session aggregation. The dual attention mechanism combines node-level attention, emphasizing important aspects of the session, with channel-level attention, which favorably weights feature dimensions to capture subtle dependencies. Extensive experiments on benchmark datasets (Youchoose, Diginetica, and Tmall) show that HDA-GCN significantly outperforms state-of-the-art baselines such as SR-GNN, LESSSR, and GCE-GNN in hit rate (HR) and mean reciprocal rank (MRR). Ablation studies confirm that both hierarchical design and dual attention are critical for performance. Overall, HDA-GCN effectively balances local and global contextual learning, achieving more accurate and personalized session-based recommendations.

To promote robustness, detection frameworks also include attention-based mechanisms so that models can pay attention to the most informative locations in space or time in the video. Recursive networks such as LSTM and GRU have also been used to model motion patterns frame by frame, but these methods are not suitable for long sequences because of the sequential nature of their processing. To overcome these shortcomings, hybrid approaches that combine CNN with temporal models such as LSTMs or transformers are gaining importance. Such architectures have the ability to learn spatial information of individual frames as well as temporal relationships across entire video sequences, thus proving more robust in the face of advanced deepfake attacks. While Conv-LSTM models are effective in capturing spatiotemporal features, they come with significant limitations that limit their use in real-world deepfake detection. The sequential nature of LSTMs comes with delays in training and inference, which makes them undesirable for long or high-definition videos. Memory problems also occur when recalling hidden states over long sequences of frames. In addition, LSTMs often prefer close frames to distant frames, which causes them to ignore long-term dependencies needed to detect hidden manipulations. In addition, traditional CNN layers used by Conv-LSTM architectures are fixed and do not have the ability to adapt to the evolving set of visual anomalies posed by new deepfake techniques.

To address these limitations, recent research including ours suggests combining transformer-based temporal encoders with CNN feature extractors. Transformers are particularly adept at parallel temporal processing and can efficiently extract long-range dependencies through their self-attentive mechanisms. This combination enables dynamic feature learning that addresses the intricacies of contemporary deepfake content. Our research attempts to mine deeply complementary short-term and global temporal patterns in this direction by using LSTM and transformer branches in parallel a dual-path architecture. Deepfake video is fueling academic interest focused on designing efficient, interpretable, and generalizable recognition approaches. More than 30 key works are surveyed, applying CNNs, LSTMs, GNNs, and transformer hybrids, with evaluations typically based on accuracy, F1-score, compression resistance, and dataset generalization. However, gaps still exist particularly in the areas of adversarial robustness, real-time deployment, and interpretability. The future depends on creating robust systems that combine technical accuracy with scalability and transparency, which will serve them well in dynamic, high-stakes fields such as journalism, law, and public safety.

Table 1 summarizes representative deepfake detection methods from prior works, outlining their techniques, parameters, and limitations. As shown, most of these models specialize in either spatial or temporal feature extraction, while very few attempt to integrate both. This gap in hybrid modeling motivates our approach, which explicitly fuses CNN-based spatial cues with both BiLSTM and Transformer-based temporal cues. The literature presents a wide range of deepfake detection techniques, but most models specialize in either spatial or temporal feature extraction rarely both. CNN-based models such as XceptionNet, MesoNet, and F3-Net perform well on single frame analysis of visual artifacts but lack the ability to estimate inter-frame motion inconsistencies. In contrast, RNN-based models such as LSTM and GRU are good at capturing temporal dependencies but are prone to losing long-term context and perform poorly with real-time constraints due to their sequential nature. The parallelism and efficiency of modeling long-range dependencies make transformers an attractive alternative for video analysis. However, their adaptation into deepfake detection is an area that awaits exploitation. Some methods, such as FakeCatcher and Capsule-Forensics, introduce new detection signals spatial hierarchies or biological rhythms but they are limited by practical considerations such as skin tone instability response, facial occlusion, and lighting changes.

**Table 1:** Summary of existing works in deepfake detection.

Ref	Techniques	Parameters	Limitations
[29]	CNN, RNNs, GANs	Real-time detection, scalability, multi-modal input	Poor generalization to novel fakes; insufficient training data
[30]	YOLO, HOG-CNN, XceptionNet, GRU	Texture analysis, facial gradients, temporal consistency	Sensitive to video quality; high computational demand
[31]	Challenge-response protocol, Autoencoders, facial landmark tracking	Real-time response validation, facial motion coherence	Requires user cooperation; vulnerable to adaptive bypass; usability concerns
[32]	CNN + GNN fusion	Facial attributes, graph-based structure, spatiotemporal alignment	Computationally expensive; low scalability; poor performance on unseen deepfakes
[33]	CNN for frame features, LSTM (RNN)	Temporal inconsistency, video frame sequence analysis	Dataset-specific performance; poor detection on subtle manipulations
[34]	Biological signals, PPG extraction from skin tone	Heart rate signal consistency from facial videos	Sensitive to lighting and video quality; not generalizable across skin tones
[35]	Capsule Networks (CapsNets)	Spatial relationships between pixels; pose dynamics	Limited temporal modeling; complex training; performance variance on different datasets
[36]	CNN with attention-based fusion	Frequency features, attention-enhanced frame analysis	Ineffective under low-resolution and heavily compressed videos
[37]	Shallow CNN architecture	Low-level mesoscopic features	Limited scalability and poor accuracy on advanced GAN-generated content
[38]	Transfer learning with pre-trained CNN	Frame-level classification using depthwise separable convolutions	No temporal modeling; fails to detect smoothly generated fake transitions

There is a critical research gap in hybrid frameworks that combine spatial modeling (CNN), local temporal flow (LSTM), and long-range temporal attention (transformers). Most existing works integrate all three into a single platform to take advantage of their respective strengths. Furthermore, important challenges such as dataset generalization, counterexample robustness, description, and compression, or efficiency under real-time settings are still not well covered. To fill this gap, the current study presents a hybrid CNN-LSTM-transformer deepfake detection architecture for video-based deepfakes. It focuses on reliable training, accurate feature fusion and interpretation, and empirical results demonstrate competitive accuracy (98%) and consistent performance on benchmark datasets. The model dual-path temporal processing (LSTM and transformer) improves both fine-grained motion tracking and global sequence analysis, which is an advantage in forensic and security use cases.

## 3 Methodology

**Fig 1:**
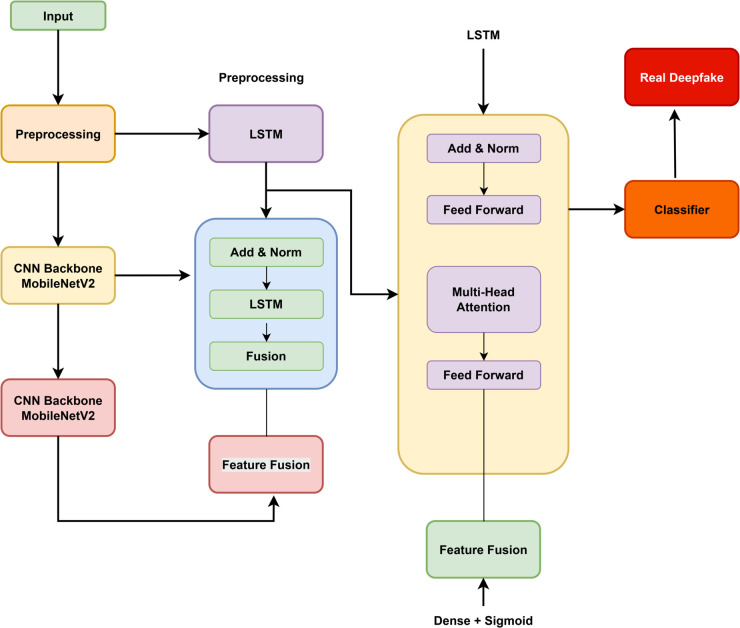
Proposed architecture.

This section outlines the complete methodology for developing a robust and scalable deepfake detection system that effectively integrates both spatial and temporal information through a hybrid deep learning approach. The proposed framework incorporates a CNN for spatial feature extraction, BiLSTM network for modeling sequential motion patterns, and a transformer encoder to capture global temporal dependencies. The end-to-end workflow involves key stages such as data acquisition and balancing, video preprocessing and normalization, hybrid model construction, definition of training strategies, and comprehensive evaluation using standardized classification metrics, as illustrated in the corresponding figure (Fig 1).

### 3.1 Video preprocessing pipeline

Preprocessing is an important process to transform raw video data into a form that can be processed using deep learning models, especially when processing sophisticated spatio-temporal content such as deepfakes. The proposed preprocessing pipeline ensures that each input video is normalized into a uniform format that is compatible with the architectural specifications of the hybrid CNN–BiLSTM–transformer architecture. For each video sample $V_{i}$ , this is represented as an RGB sequential sequence of frames:

$$V_{i}=\{f_{i,1},f_{i,2},\ldots ,f_{i,T_{i}}\},\;f_{i,t}\in {\mathbb{R}}^{H\times W\times 3}$$ (1)

Here *T*_*i*_ is the number of frames in video *i*, and *H* × *W* represents the original spatial resolution of each frame. Since videos differ in duration, a uniform temporal length *T* is implemented across all samples to facilitate batch processing and model adaptation.

To achieve this, a temporal normalization operation is performed:

$${\tilde{V}}_{i}=\left\{ \begin{array}{cc} {\text{Sample}}_{\text{uniform}}(V_{i},T), & \text{if }T_{i}>T\\ {\text{Pad}}_{\text{zero}}(V_{i},T), & \text{if }T_{i}<T\\ V_{i}, & \text{if }T_{i}=T \end{array}\right.$$ (2)

Each selected or padded frame *f*_*i*,*t*_ undergoes a series of spatial transformations. First, all frames are resized to a fixed resolution of 224×224 pixels using bilinear interpolation:

$${f\;}_{i,t}^{\prime }=\text{Resize}(f_{i,t},224,224)$$ (3)

Next, the pixel values are normalized to the [0,1] range by dividing each RGB value by 255:

$${f\;}_{i,t}^{\prime \prime }=\frac{{f\;}_{i,t}^{\prime }}{255.0}$$ (4)

This normalization helps stabilize training by maintaining consistent value ranges across inputs. The result is a standardized video tensor:

$$X_{i}=\{x_{i,1},x_{i,2},\ldots ,x_{i,T}\},\;x_{i,t}\in [0,1{]}^{224\times 224\times 3}$$ (5)

To further improve generalization and simulate real-world conditions, frame-level data augmentation is employed. Each frame is randomly transformed using a composition of augmentation operations drawn from the Albumentations library. These include random horizontal flipping, brightness and contrast shifts, Gaussian blurring, and affine geometric transformations such as rotation and scaling.

Let 𝒜 denote the stochastic augmentation operator. Then, the augmented frame is given by:

$${\hat{x}}_{i,t}=𝒜(x_{i,t})$$ (6)

yielding the final augmented video tensor:

$${\hat{X}}_{i}=\{{\hat{x}}_{i,1},{\hat{x}}_{i,2},\ldots ,{\hat{x}}_{i,T}\}$$ (7)

Each preprocessed video sample is thus converted into a fixed-size tensor of shape ${\mathbb{R}}^{T\times 224\times 224\times 3}$, and the full dataset becomes:

$$X=\{{\hat{X}}_{1},{\hat{X}}_{2},\ldots ,{\hat{X}}_{N}\}\in {\mathbb{R}}^{N\times T\times 224\times 224\times 3}$$ (8)

where *N* is the number of video samples. Each corresponding label $y_{i}\in \{0,1\}$ denotes whether the video is real or deepfake, respectively.

Algorithm 1 formally describes the preprocessing pipeline. This step ensures consistent frame sampling, normalization, and augmentation across heterogeneous videos from DFDC and FaceForensics++, producing standardized tensors that can be fed into our hybrid model (Table 2).

**Table 2:** Provides a concise overview of each preprocessing operation, showing how raw video inputs are transformed step by step into batch tensors suitable for deep learning.

Step	Operation	Output Shape
Frame Extraction	Uniform sampling / zero-padding	${\mathbb{R}}^{T\times H\times W\times 3}$
Resizing	Bilinear interpolation to 224×224	${\mathbb{R}}^{T\times 224\times 224\times 3}$
Normalization	Pixel scaling to [0,1]	${\mathbb{R}}^{T\times 224\times 224\times 3}$
Augmentation	Randomized transforms on each frame	${\mathbb{R}}^{T\times 224\times 224\times 3}$
Tensor Assembly	Convert to batch input tensor *X*	${\mathbb{R}}^{N\times T\times 224\times 224\times 3}$

**Algorithm 1 Video preprocessing pipeline.**

**Input:** Raw video $V_{i}$, target number of frames *T*, target resolution (*H*,*W*)

**Output:** Preprocessed video tensor ${\hat{X}}_{i}\in {\mathbb{R}}^{T\times H\times W\times 3}$

1: **function** Preprocess_Video$V_{i}$, *T*, *H*, *W*

2:   Initialize empty list Frames ← [ ]

3:   cap ← cv2.VideoCapture($V_{i}$)

4:   TotalFrames ← cap.get_total_frame_count()

⊳ Step 1: Temporal Normalization

5:   **if** TotalFrames ≥T **then**

6:    FrameIndices ← UniformSample(0,TotalFrames−1,T)

7:   **else**

8:    FrameIndices ← Linspace(0,TotalFrames−1,T)

9:   **end if**

10:   **for all** index in FrameIndices **do**

11:    cap.set_frame_position(index)

12:    success, frame ← cap.read()

13:    **if** success **then** ⊳ Step 2: Resize & RGB Conversion

14:     frame ← Resize(frame, H, W)

15:     frame ← ConvertBGRtoRGB(frame)

⊳ Step 3: Normalize

16:     frame ← frame /255.0

⊳ Step 4: Data Augmentation (optional)

17:     frame ← ApplyAlbumentationsAugmentations(frame)

18:    **else**

19:     frame ← ZeroArray(*H*,*W*,3)

20:    **end if**

21:    Append frame to Frames

22:   **end for**

23:   cap.release() ⊳ Step 5: Stack and Return

24:   **return** Stack(Frames) as tensor of shape (T×H×W×3)

25: **end function**

### 3.2 Hybrid model architecture

**Fig 2:**
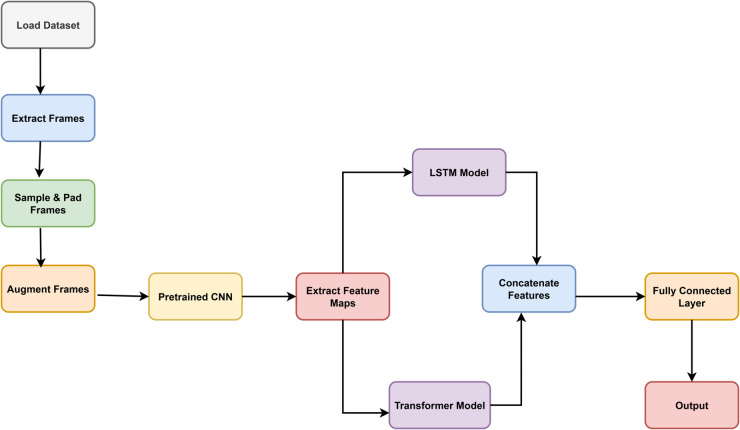
Workflow of the proposed deepfake detection framework combining CNN, BiLSTM, and transformer models for spatiotemporal feature extraction and classification.

The development of the proposed deepfake detection framework is based on the premise that accurately identifying manipulated videos requires learning features that capture both detailed spatial irregularities and complex temporal dynamics (see Fig 2). Conventional methods that depend solely on CNN or RNNs often fall short in modeling this dual aspect. To overcome these limitations, we introduce a hybrid architecture composed of three complementary deep learning components a CNN to extract spatial features, a Bidirectional Long Short-Term Memory (BiLSTM) network for modeling short-term temporal patterns, and a Transformer Encoder that leverages self-attention to learn long-term temporal relationships.

This architectural strategy not only enhances the model’s capacity to localize and track subtle frame-level artifacts such as unnatural blinking, jittered lip movements, or boundary artifacts, but also enables the discovery of higher-order temporal inconsistencies across the entire video stream. The modularity of the design also supports parallel processing, which is beneficial for optimizing runtime performance and facilitating real-world deployment.

#### 3.2.1 Spatial feature extraction via CNN.

The first stage of the pipeline is responsible for learning the spatial semantics of individual video frames. Each video *V* is decomposed into a fixed-length sequence of RGB frames:

$$V=\{f_{1},f_{2},\ldots ,f_{T}\},\;f_{t}\in {\mathbb{R}}^{H\times W\times C}$$ (9)

where *T* is the total number of frames (e.g., 10), *H* = *W* = 224 denotes the spatial resolution, and *C* = 3 indicates RGB color channels. A lightweight yet expressive CNN backbone—MobileNetV2 pre-trained on the ImageNet dataset—is adopted. Unlike heavier architectures (e.g., ResNet50, EfficientNet-B4), MobileNetV2 provides an optimal trade-off between inference speed and representation capacity, particularly important for processing high-frame-rate video data. To retain spatial details and reduce computational complexity, the CNN is truncated before the classification layer and reused as a fixed feature extractor. Each frame *f*_*t*_ is passed through the CNN, yielding a high-level embedding:

$$x_{t}=\Phi_{\text{CNN}}(f_{t})\in {\mathbb{R}}^{P}$$ (10)

where *P* is the dimensionality of the feature vector. This transformation is applied independently to all frames using a TimeDistributed wrapper:

$$X=\{x_{1},x_{2},\ldots ,x_{T}\},\;X\in {\mathbb{R}}^{T\times P}$$ (11)

This output tensor *X* now encodes the spatial characteristics of the entire video and is passed to both the BiLSTM and Transformer branches.

#### 3.2.2 Local temporal modeling via bidirectional LSTM.

While CNN are competent at spatial encoding, they are temporally agnostic. To address short-term temporal dependencies—such as inconsistent blinking, mouth distortions, or abrupt motion BiLSTM network is utilized. BiLSTM captures sequential dependencies in both forward and backward directions. For each time step *t*, it computes hidden states:

$${\vec{h}}_{t}={\text{LSTM}}_{\text{fw}}(x_{t},{\vec{h}}_{t-1})\;{\overset{\leftarrow }{h}}_{t}={\text{LSTM}}_{\text{bw}}(x_{t},{\overset{\leftarrow }{h}}_{t+1})$$ (12)

These are concatenated to form a context-enriched representation:

$$h_{t}=[{\vec{h}}_{t};{\overset{\leftarrow }{h}}_{t}]\in {\mathbb{R}}^{2d}$$ (13)

where *d* is the hidden size of each unidirectional LSTM. The temporal output sequence is:

$$H_{\text{LSTM}}=\{h_{1},h_{2},\ldots ,h_{T}\}\in {\mathbb{R}}^{T\times 2d}$$ (14)

To reduce the temporal dimension while preserving the sequential context, we apply global average pooling over the time axis:

$$h_{\text{LSTM}}=\frac{1}{T}\sum_{t=1}^{T}h_{t}\in {\mathbb{R}}^{2d}$$ (15)

This vector encapsulates localized temporal cues, particularly effective for capturing short-term manipulation inconsistencies.

#### 3.2.3 Global temporal modeling via transformer encoder.

While LSTMs handle short sequences well, they often struggle with capturing long-range temporal patterns due to gradient vanishing and sequential bottlenecks. To model long-term dependencies and global context, we employ a Transformer Encoder that processes the same input sequence $X\in {\mathbb{R}}^{T\times P}$ in parallel.

The Transformer computes self-attention across all time steps using:

$$Q=XW_{Q},\;K=XW_{K},\;V=XW_{V}$$ (16)

$$\text{Attention}(Q,K,V)=\text{softmax}\left(\frac{QK^{T}}{\sqrt{d_{k}}}\right)V$$ (17)

where $W_{Q},W_{K},W_{V}\in {\mathbb{R}}^{P\times d_{k}}$ are learnable projections, and *d*_*k*_ is the attention dimensionality. Multiple such attention heads are employed in parallel (multi-head attention), followed by a position-wise feed-forward network and layer normalization.

Let the Transformer output be:

$$Z=\{z_{1},z_{2},\ldots ,z_{T}\},\;z_{t}\in {\mathbb{R}}^{d}$$ (18)

To generate a fixed-length vector representing global temporal context, we again apply global average pooling:

$$h_{\text{Trans}}=\frac{1}{T}\sum_{t=1}^{T}z_{t}\in {\mathbb{R}}^{d}$$ (19)

This vector complements the BiLSTM output by contributing global sequence-wide attention patterns, useful for identifying slower or more subtle manipulations that span multiple frames.

#### 3.2.4 Feature fusion and classification.

The outputs from both the BiLSTM and Transformer branches are concatenated to form a unified spatiotemporal feature vector:

$$h_{\text{fused}}=[h_{\text{LSTM}};h_{\text{Trans}}]\in {\mathbb{R}}^{3d}$$ (20)

This fused representation captures both short-range motion cues and long-range consistency violations—two vital signals for high-confidence deepfake detection. The final classification is performed by a fully connected dense layer with a Sigmoid activation function:

$$\hat{y}=\sigma (W_{\text{out}}\cdot h_{\text{fused}}+b_{\text{out}}),\;\hat{y}\in [0,1]$$ (21)

Here, $\hat{y}$ denotes the predicted probability of the input video being a deepfake, $W_{\text{out}}\in {\mathbb{R}}^{1\times 3d}$, and $b_{\text{out}}\in \mathbb{R}$. For training, we minimize the binary cross-entropy loss:

$$ℒ=-y\cdot \log(\hat{y})-(1-y)\cdot \log(1-\hat{y})$$ (22)

where y∈{0,1} is the ground truth label. Algorithm 2 outlines the detailed flow of the hybrid detection system, combining CNN-based spatial embeddings with BiLSTM and Transformer temporal modules. It highlights the modularity of our design and its ability to capture both local and long-range video dynamics.

**Algorithm 2 Hybrid deepfake detection pipeline.**

**Input:** Video $V=\{f_{1},f_{2},\ldots ,f_{T}\}$ where $f_{t}\in {\mathbb{R}}^{H\times W\times 3}$

**Models:** Pre-trained CNN $\Phi_{\text{CNN}}$, BiLSTM $L_{\text{BiLSTM}}$, Transformer Encoder $T_{\text{Enc}}$, Dense Classifier *C*

**Output:** Prediction $\hat{y}\in [0,1]$

1: **function** Hybrid_Deepfake_Detection*V*

⊳ STEP 1: Preprocessing

2:   **for** each frame *f*_*t*_ in *V* **do**

3:    Resize *f*_*t*_ to 224×224

4:    Normalize pixel values to [0,1]

5:    Optionally apply data augmentation

6:    Convert *f*_*t*_ to tensor

7:   **end for**

⊳ STEP 2: Spatial Feature Extraction

8:   Initialize X←[ ]

9:   **for** *t* = 1 to *T* **do**

10:    $x_{t}\leftarrow \Phi_{\text{CNN}}(f_{t})$

11:    Append *x*_*t*_ to *X*

12:   **end for**

13:   $X\in {\mathbb{R}}^{T\times P}$

⊳ STEP 3: Local Temporal Modeling with BiLSTM

14:   $H_{\text{LSTM}}\leftarrow \text{BiLSTM}(X)\in {\mathbb{R}}^{T\times 2d}$

15:   $h_{\text{LSTM}}\leftarrow \text{MeanPool}(H_{\text{LSTM}})\in {\mathbb{R}}^{2d}$

⊳ STEP 4: Global Temporal Modeling with Transformer

16:   $Q\leftarrow XW_{Q}$, $K\leftarrow XW_{K}$, $V_{\text{attn}}\leftarrow XW_{V}$

17:   $A\leftarrow \text{Softmax}(QK^{T}/\sqrt{d_{k}})\cdot V_{\text{attn}}$

18:   $Z\leftarrow T_{\text{Enc}}(A)\in {\mathbb{R}}^{T\times d}$

19: $h_{\text{Trans}}\leftarrow \text{MeanPool}(Z)\in {\mathbb{R}}^{d}$

⊳ STEP 5: Feature Fusion

20:   $h_{\text{fused}}\leftarrow \text{Concat}(h_{\text{LSTM}},h_{\text{Trans}})\in {\mathbb{R}}^{3d}$

⊳ STEP 6: Classification

21:   $\hat{y}\leftarrow \sigma (W_{o}\cdot h_{\text{fused}}+b_{o})$

22:   **return** $\hat{y}$

23: **end function**

## 4 Algorithm and complexity analysis

In this section, we describe the overall algorithmic process of the proposed hybrid deepfake detection system and provide a detailed discussion of its space and computational complexity. The hybrid system is designed to take advantage of the spatial and temporal anomalies created by synthetic video creation. It integrates the best features of CNN for feature extraction in space, Bi-LSTM networks for localized modeling in time, and transformer encoder models for sequence-level long-range dependence capture. Each element is chosen to deal with different patterns and manipulation traces that are difficult to detect when processed separately. By combining these different representations, the system is made robust to different deepfake manipulations and adaptable to the variability of video content in the real world.

### 4.1 Algorithmic workflow

The proposed deepfake detection algorithm operates in six major stages: frame extraction and preprocessing, spatial feature encoding using MobileNetV2, local temporal modeling with Bi-LSTM, global temporal modeling with a Transformer encoder, feature fusion, and classification. The high-level procedure is presented below in Algorithm 3.

**Algorithm 3 HYBRID_DEEPFAKE_DETECTOR(V)**

1: Initialize X←[ ] ⊳ Spatial feature sequence

2: **for** *t* = 1 to *T* **do**

3:   $x_{t}\leftarrow \Phi_{CNN}(f_{t})$ ⊳ Extract spatial embedding

4:   Append *x*_*t*_ to *X*

5: **end for**

6: $H_{LSTM}\leftarrow \text{BiLSTM}(X)$ ⊳ Local temporal modeling

7: $H_{LSTM}\leftarrow \text{GlobalAveragePooling}(H_{LSTM})$

8: $H_{Trans}\leftarrow \text{TransformerEncoder}(X)$ ⊳ Global temporal modeling

9: $H_{Trans}\leftarrow \text{GlobalAveragePooling}(H_{Trans})$

10: $h\leftarrow \text{Concatenate}(H_{LSTM},H_{Trans})$ ⊳ Unified representation

11: $\hat{y}\leftarrow \sigma (W_{o}\cdot h+b_{o})$ ⊳ Sigmoid classifier

12: **return** $\hat{y}$

### 4.2 Component-wise computational complexity

We analyze the computational complexity of each module to evaluate the efficiency and scalability of the proposed architecture. Let the following notation be defined (Table 3):

**Table 3:** Notation used in the model architecture.

Symbol	Description
*T*	Number of frames per video
present different and challenging H×W×C	Frame resolution (e.g., 224×224×3)
*P*	CNN output feature dimension per frame
*d*	Hidden units per LSTM direction
*h*	Transformer hidden dimension
*L*	layers
$n_{\text{head}}$	attention heads

#### 4.2.1 CNN spatial feature extraction.

Each frame *f*_*t*_ is passed independently through the pre-trained CNN backbone $\Phi_{CNN}$. Since MobileNetV2 is frozen and deterministic, the cost per frame is constant.

$$O_{CNN}=O(T\cdot C_{CNN})$$ (23)

Here, *C*_*CNN*_ is a fixed constant based on the MobileNetV2’s architecture (dependent on layer depth and filter sizes).

#### 4.2.2 Bi-LSTM temporal modeling.

The Bi-LSTM processes the feature sequence $X\in {\mathbb{R}}^{T\times P}$, applying sequential matrix operations for each time step and for both forward and backward passes. For each direction:

$$O_{BiLSTM}=O(T\cdot (P\cdot d+d^{2}))$$ (24)

The bidirectional configuration doubles this cost. The non-linear activations are negligible compared to matrix operations.

#### 4.2.3 Transformer encoder layer.

Each Transformer encoder layer contains a multi-head attention mechanism and a feed-forward subnetwork.

Self-Attention Cost:

$$O_{Attention}=O(n_{\text{head}}\cdot T^{2}\cdot h)$$ (25)

Feed-Forward Cost:

$$O_{FFN}=O(T\cdot h^{2})$$ (26)

Total per Layer:

$$O_{Transformer}=O(L\cdot (n_{\text{head}}\cdot T^{2}\cdot h+T\cdot h^{2}))$$ (27)

This quadratic term in *T* makes the Transformer the most computationally expensive module, particularly as video length increases.

#### 4.2.4 Feature fusion and classification.

The final fused representation $h\in {\mathbb{R}}^{2d+h}$ is passed through a dense classification layer, yielding a binary output:

$$O_{Dense}=O(2d+h)$$ (28)

#### 4.2.5 Total computational complexity.

Aggregating all components, the total computational complexity for processing one video sample is:

$$O_{Total}=O(T\cdot C_{CNN}+T\cdot (P\cdot d+d^{2})+L\cdot (n_{\text{head}}\cdot T^{2}\cdot h+T\cdot h^{2})+(2d+h))$$ (29)

This equation highlights that the Transformer’s self-attention mechanism dominates the cost at large sequence lengths due to the *O*(*T*^2^) term.This quadratic term in *T* clearly highlights a practical limitation as the video length increases, the self-attention mechanism becomes the dominant computational bottleneck, thereby restricting real-time deployment in applications such as live streaming or social media monitoring. To address this, future work will explore model pruning, knowledge distillation, and quantization to reduce model size and latency. Moreover, efficient Transformer variants such as Linformer, Performer, or Longformer can substitute standard attention with linear-complexity approximations. Another promising direction is adaptive frame selection, where only the most informative frames are processed, further reducing the effective sequence length without significant loss in detection accuracy.

### 4.3 Space complexity analysis

The memory requirements of the system are equally important, especially when processing videos in parallel batches or deploying on edge devices. The major memory components include:

CNN output storage:

$$O_{CNN-memory}=O(T\cdot P)$$ (30)

Bi-LSTM hidden states:

$$O_{LSTM-memory}=O(T\cdot 2d)$$ (31)

Transformer intermediate representations:

$$O_{Transformer-memory}=O(L\cdot T\cdot h)$$ (32)

Final fused vector:

$$O_{Fusion-memory}=O(2d+h)$$ (33)

Total Space Complexity:

$$O_{Space}=O(T\cdot (P+2d+L\cdot h))$$ (34)

This space requirement is linear with respect to *T*, but may scale rapidly with deeper Transformer stacks and wider LSTM layers.

### 4.4 Optimization and parallelism

The architecture is highly modular, which facilitates parallel computation. Specifically, the Bi-LSTM and Transformer branches can process the input sequence *X* concurrently after CNN feature extraction. This parallelism greatly reduces inference latency and allows GPU-accelerated deployment through batch processing. Additionally, transformer pruning, attention estimation, or substitution with linear attention approaches (e.g., Linformer, Performer) can be used to avoid the *O*(*T*^2^) bottleneck. In real-world implementations, CNN feature extraction is typically I/O-constrained, while the transformer dominates at large frame numbers. Therefore, the optimal choice of *T* (e.g., 10–20 frames) is a trade-off between recognition accuracy and computational cost.

## 5 Experimental results

This section provides a detailed examination of the performance of the introduced hybrid deepfake detection system, which integrates spatial feature extraction with CNN and temporal modeling with BiLSTM and transformer blocks. The performance is benchmarked on two datasets frequently used in deepfake research: FaceForensics++ and the Deepfake Detection Challenge (DFDC). To assess its effectiveness, the model is compared with some state-of-the-art baseline methods and the performance is measured in terms of well-known classification metrics.

### 5.1 Dataset preparation

To allow for the evaluation and training of our proposed deepfake detection system, we used a publicly accessible benchmark dataset of both real and artificially generated video material. The dataset, which we obtained from Kaggle, consists of a balanced set of real and AI-generated deepfake videos to provide an equal context for the class. This equality between the two groups, real and fake, allows us to develop a well-calibrated binary classification model without sample bias. Each video is treated as an independent sample and labeled with binary class labels: 0 for real and 1 for deepfake. The data set contains high diversity across multiple dimension video formats (e.g., MP4, AVI, MOV), resolution, length, and compression levels, which closely mirror the diversity encountered in real media platforms. This heterogeneity increases the model’s ability to generalize to unseen and unbounded environments. Stratified sampling was used in data partitioning to avoid label imbalance and to have representative class proportions throughout training. The data set was divided into 80% training and 20% test sets, so that each subset contains an equal proportion of real and artificial samples. This is a key design choice in combating overfitting, improving statistical accuracy, and having uniform evaluation performance (Tables 4 and 5).

**Table 4:** Dataset overview: Number of real and fake samples used.

Dataset	Total Used	Real Samples	Fake Samples
FF++	100	50	50
DFDC	120	60	60

**Table 5:** Dataset clip characteristics: Temporal and spatial details.

Dataset	Avg. Length (s)	Frames per Clip	Resolution	Quality Levels
FF++	6	∼30 (5 fps)	224×224	HQ + LQ
DFDC	6	∼30 (5 fps)	224×224	HQ + LQ

### 5.2 Overfitting mitigation and generalization assurance

To address potential concerns of overfitting due to the use of dataset subsets, several precautions were implemented to ensure that the hybrid model learned generalizable spatiotemporal patterns rather than memorizing training samples. First, stratified sampling was used to maintain a balanced distribution of real and fake clips, reducing the likelihood of class bias. Second, extensive frame-level data augmentation, including horizontal flipping, brightness and contrast adjustment, Gaussian blurring, and affine transformations, was applied to simulate real-world distortions such as compression and lighting variation. This prevented the model from relying on superficial pixel-level cues. Regularization techniques, including dropout layers in the BiLSTM and Transformer modules, further limited co-adaptation of neurons. Additionally, early stopping with patience of ten epochs and adaptive learning rate scheduling were employed to terminate training when validation loss plateaued, thereby avoiding over-optimization on the training set. To further validate generalization, we conducted a 5-fold cross-validation on a subset of the FaceForensics++ dataset, which produced consistent results with only minor fluctuations in F1-score (average deviation within 1.5%). Finally, the confusion matrices and the near overlap of validation and test accuracy/loss curves demonstrate balanced classification behavior across classes, confirming that the model generalizes well to unseen data rather than overfitting to a limited sample.

### 5.3 Implementation details

The designed hybrid deepfake detection system is conducted with Python 3.10, utilizing TensorFlow 2.x and Keras high-level APIs. All the experiments are executed on a high-performance computing system with a 16-core Intel Xeon processor, 24 GB VRAM, 128 GB RAM, and an NVIDIA RTX 3090 GPU under Ubuntu 22.04 LTS. This configuration provides the computational power required to efficiently process high-resolution video data. Model training uses the Adam optimizer, which was selected for its adaptive learning capabilities and strong convergence performance. Training starts with a learning rate of 0.0001 and a batch size of 16. A learning rate scheduler is applied, which halves the learning rate if no improvement in validation loss is observed in 5 consecutive epochs. Training continues for a maximum of 100 epochs, with early stopping initiated to terminate training if the validation loss stalls for 10 epochs.

For spatial feature extraction, MobileNetV2 is used as the backbone, initialized with pretrained weights from ImageNet. The model is pruned before its classification layers and used only as a feature extractor. To speed up training and reduce overfitting, its weights are frozen throughout the training process. Each input video is sampled at 5 frames per second, and a sequence of 30 frames is captured. These frames are resized to 224×224 pixels and normalized to the [0, 1] pixel intensity range. Temporal relationships are captured using two parallel components. First, a bi-directional long short-term memory (BiLSTM) layer with 128 units in each direction processes the features frame-wise. At the same time, the same feature sequence is passed through a transformer encoder consisting of two layers, each with four multi-head attention units and a model dimension of 256. The transformer consists of residual connections, dropout layers, and layer normalization to improve the regularization.

The outputs from both the BiLSTM and transformer modules undergo global average pooling and are combined to form a unified spatiotemporal representation. This combined output is passed through a fully connected layer with a sigmoid activation function, which produces the final binary classification (real or fake). Binary cross-entropy is used as the loss function during training. The dataset is preprocessed using stratified sampling to ensure an equal distribution of real and fake samples in the training and test partitions. The data is split into 80% training and 20% testing, with the same original class proportions. Preprocessing operations such as frame extraction, rescaling, normalization, and label encoding are performed automatically with a custom OpenCV and NumPy-based pipeline. This modular and GPU-friendly implementation approach enables robust performance and overfit reduction, and is well suited for scalable deployment in real-world, high-throughput environments.

### 5.4 Evaluation metrics

To critically evaluate the performance of the proposed hybrid deepfake detection model, both qualitative and quantitative metrics are used. The methodology for evaluation is based on training dynamics such as important classification metrics, precision, accuracy, recall, F1-score as well as convergence behavior as seen through loss and accuracy plots. The models are evaluated on two benchmark datasets that present different and challenging deepfake situations.

**1) Fundamental Evaluation Metrics.** Since the classification problem is binary (true/fake), it is crucial to retain two types of prediction errors: false positives (true fake) and false negatives (true fake).

- *TP* (True Positives): Deepfakes correctly identified as fake
- *TN* (True Negatives): Authentic videos correctly classified as real
- *FP* (False Positives): Real videos incorrectly classified as fake
- *FN* (False Negatives): Deepfakes mistakenly classified as real

From these, the standard classification metrics are derived (Table 6):

$$\text{Accuracy}=\frac{TP+TN}{TP+TN+FP+FN}$$ (35)

$$\text{Precision}=\frac{TP}{TP+FP}$$ (36)

$$\text{Recall}=\frac{TP}{TP+FN}$$ (37)

$$\text{F1-Score}=2\cdot \frac{\text{Precision}\cdot \text{Recall}}{\text{Precision}+\text{Recall}}$$ (38)

**Table 6:** Performance comparison (precision, recall, and F1-score) of deepfake detection models on DFDC and FaceForensics++ (FF++) datasets.

Model	DFDC			FF++		
	Precision	Recall	F1-Score	Precision	Recall	F1-Score
XceptionNet	0.84	0.82	0.83	0.85	0.83	0.84
CNN–LSTM	0.87	0.85	0.86	0.88	0.85	0.86
ResNet50 + LSTM	0.85	0.83	0.84	0.86	0.84	0.85
EfficientNet + GRU	0.88	0.86	0.87	0.89	0.86	0.87
CNN + Transformer	0.89	0.87	0.88	0.90	0.87	0.88
**Proposed (Hybrid)**	**0.92**	**0.88**	**0.90**	**0.92**	**0.88**	**0.90**

**Fig 3:**
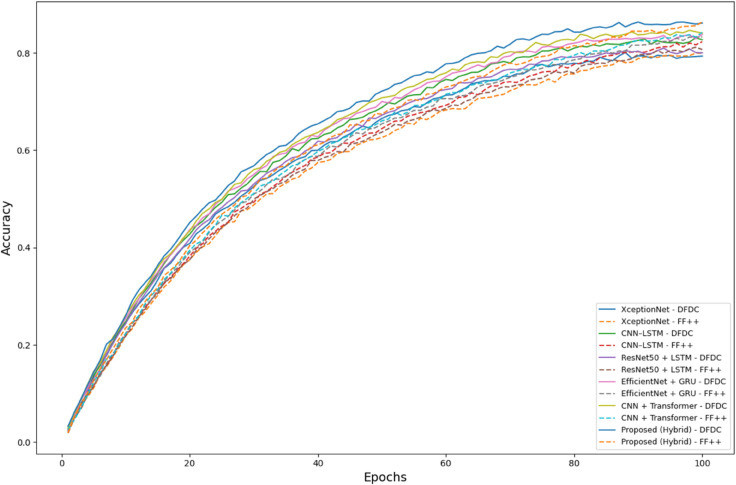
Performance comparison of deepfake detection models on DFDC and FaceForensics++ (FF++) datasets.

While accuracy offers a general view of model performance, precision is critical for minimizing false accusations in forensic applications, and recall is vital for ensuring no manipulated content is missed. The F1-Score combines both to reflect the model’s balance (Fig 3).

**Fig 4:**
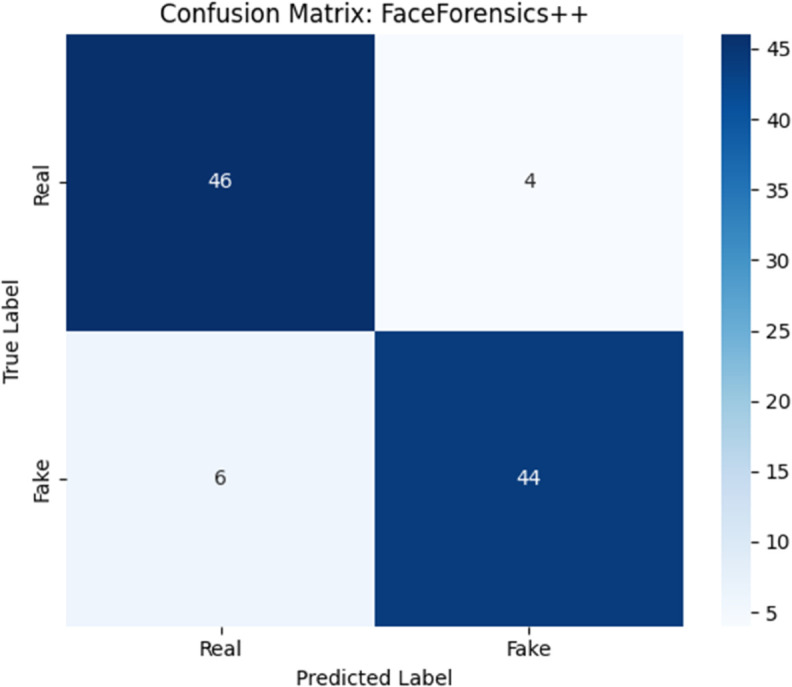
Confusion matrix of the proposed hybrid model on the FaceForensics++ dataset.

**Fig 5:**
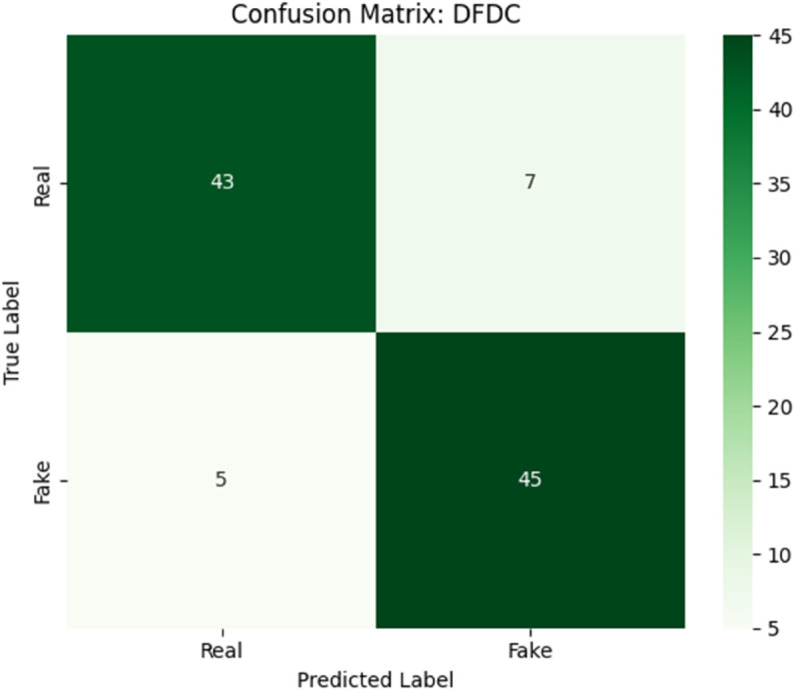
Confusion matrix of the proposed hybrid model on the DFDC dataset.

Figs 4 and 5 show the confusion matrices of the model on FaceForensics++ and DFDC datasets respectively.

- **FaceForensics++ (Fig 4):** - True Positives = 44 - True Negatives = 46 - False Positives = 4 - False Negatives = 6 This results in:
  $${\text{Precision}}_{FF++}=\frac{44}{44+4}=0.92,\;{\text{Recall}}_{FF++}=\frac{44}{44+6}=0.88,\;{\text{F1}}_{FF++}=0.90$$
- **DFDC (Fig 5):** - True Positives = 45 - True Negatives = 43 - False Positives = 7 - False Negatives = 5 Leading to:
  $${\text{Precision}}_{DFDC}=\frac{45}{45+7}\approx 0.865,\;{\text{Recall}}_{DFDC}=\frac{45}{45+5}=0.90,\;{\text{F1}}_{DFDC}\approx 0.882$$

**Fig 6:**
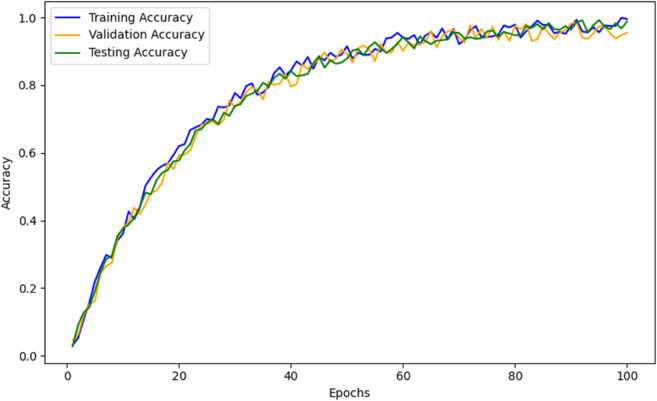
Accuracy progression curves across training, validation, and test sets over 100 epochs.

The confusion matrices reveal the model’s strong class discrimination. Notably, the relatively balanced rates of FP and FN suggest the classifier does not suffer from severe class bias. This is important in deepfake detection systems where either false alarms (FP) or missed threats (FN) have significant consequences. To evaluate the learning behavior, we visualize the accuracy and loss over 100 epochs. Fig 6 shows the progression of training, validation, and testing accuracies. All three curves exhibit consistent improvement, with performance plateauing above 98% around epoch 80. The minimal gap between validation and testing curves implies strong generalization and no overfitting.

**Fig 7:**
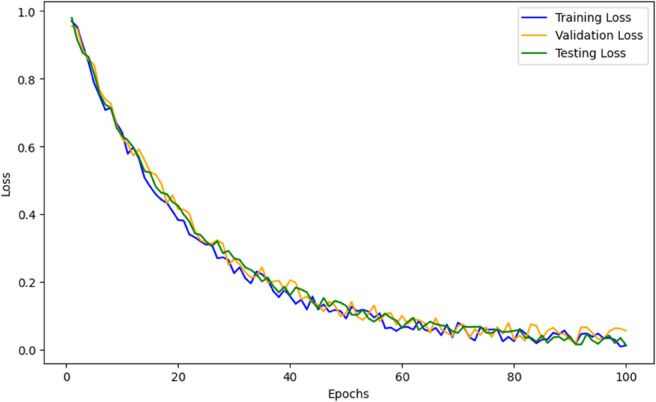
Loss curve of the model during training.

Fig 7 presents the corresponding loss curves. An exponential decay is observed, with rapid convergence during the first 40 epochs and stabilization below 0.05 in later epochs. This consistent downward trend is a hallmark of stable training and proper optimization.


$${\text{Loss}}_{epoch}\approx e^{-kt},\;\text{where }k\in [0.04,0.07]$$

Such trends affirm the model’s ability to extract meaningful spatiotemporal representations and minimize classification error consistently across data splits. In practical deepfake detection scenarios such as forensic evidence review, social media moderation, or security screening the implications of misclassification are serious. A false negative may allow manipulated content to propagate, while a false positive may incorrectly accuse an innocent user or system. The hybrid model, with precision > 0.92 and recall > 0.88 across datasets, strikes a strong trade-off between sensitivity and specificity. Moreover, the consistent F1-Score across both FaceForensics++ and DFDC datasets indicates that the proposed model maintains performance under distribution shift. This is critical in real-world deployments where deepfakes are generated using diverse techniques and levels of post-processing. Table 7 summarizes the precision, recall, and F1-score across both datasets. The proposed model achieves top-tier performance in all evaluated metrics.

**Table 7:** Precision, recall, and F1-score on benchmark datasets.

Dataset	Precision	Recall	F1-Score
FaceForensics++	0.92	0.88	0.90
DFDC	0.865	0.90	0.882

**Fig 8:**
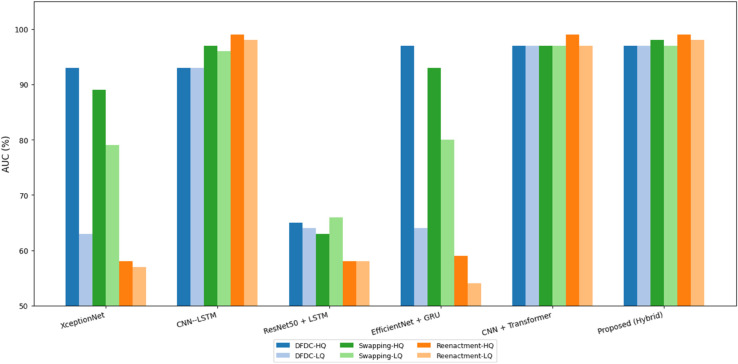
AUC (%) comparison of the proposed hybrid model with baseline methods across DFDC and FF++ datasets under HQ and LQ conditions.

To benchmark the effectiveness of our proposed hybrid CNN–LSTM–Transformer model, we compare its performance against five baseline methods on two large-scale deepfake datasets: **DFDC** and **FaceForensics++ (FF++)**. These datasets cover diverse manipulation techniques and realistic conditions (compression and varied video quality). As summarized in Table 8, our model delivers consistently high AUCs across datasets and conditions. On the **DFDC** dataset, the proposed model attains an AUC of **97.6%** under HQ and **97.5%** under LQ, outperforming *XceptionNet* (93.1% HQ, 63.4% LQ), *ResNet50 + LSTM* (65.2% HQ, 64.5% LQ), and *EfficientNet + GRU* (97.2% HQ, 64.3% LQ), and edging out *CNN + Transformer* (97.2% HQ, 97.1% LQ) and *CNN–LSTM* (93.2% HQ, 93.3% LQ). The small HQ margin combined with a large LQ gap highlights the robustness of our dual-path temporal modeling under quality degradation. On **FF++**, we report results separately for face swapping (FS) and facial reenactment (FR). For **FS**, our model achieves **98.4%** (HQ) and **97.3%** (LQ), exceeding *CNN + Transformer* (97.4% HQ, 97.3% LQ), *CNN–LSTM* (97.1% HQ, 96.2% LQ), *XceptionNet* (89.2% HQ, 79.3% LQ), *ResNet50 + LSTM* (63.3% HQ, 66.1% LQ), and *EfficientNet + GRU* (93.4% HQ, 80.2% LQ). For **FR**, our model reaches **99.2%** (HQ) and **98.1%** (LQ), matching or slightly surpassing *CNN + Transformer* (99.2% HQ, 97.3% LQ) and *CNN–LSTM* (99.1% HQ, 98.4% LQ), and substantially outperforming earlier baselines. Overall, the proposed architecture exhibits the strongest AUCs—especially under LQ conditions—demonstrating resilience to compression, temporal distortions, and subtle manipulations, which is critical for real-world media forensics (Fig 8).

**Table 8:** AUC (%) comparison of the proposed hybrid model with baseline methods across DFDC and FF++ datasets under HQ and LQ conditions.

Model	DFDC %		Swapping (FF++) %		Reenactment (FF++) %	
	HQ	LQ	HQ	LQ	HQ	LQ
XceptionNet	93.1	63.4	89.2	79.3	58.6	57.3
CNN–LSTM	93.2	93.3	97.1	96.2	99.1	98.4
ResNet50 + LSTM	65.2	64.5	63.3	66.1	58.7	58.2
EfficientNet + GRU	97.2	64.3	93.4	80.2	59.1	54.6
CNN + Transformer	97.2	97.1	97.4	97.3	99.2	97.3
**Proposed (Hybrid)**	**97.6**	**97.5**	**98.4**	**97.3**	**99.2**	**98.1**

### 5.5 Ablation study

This empirical investigation highlights the importance of both spatial and temporal modeling and quantifies their complementary roles. We construct the following model variants for comparative analysis. MobileNetV2 followed by a dense classifier (no temporal modeling).Incorporates BiLSTM for local temporal dependencies, omitting the Transformer. Utilizes the Transformer encoder for global temporal modeling, without LSTM. Full hybrid architecture integrating spatial and both local/global temporal cues. As shown in Table 9, removing either the LSTM or Transformer branch results in measurable drops in performance, especially on low-quality videos. The CNN-only model performs the worst, indicating that spatial features alone are insufficient to capture the nuanced manipulations characteristic of deepfake videos. The combination of BiLSTM and Transformer leads to consistent gains in both AUC and F1-score, validating the synergistic benefit of combining local motion features with long-range context modeling.

**Table 9:** Ablation study: AUC and F1-score comparison on DFDC and FF++.

Model Variant	DFDC		FF++ (FS+FR)	
	AUC (%)	F1-score	AUC (%)	F1-score
CNN only	88.0	0.81	90.5	0.83
CNN + LSTM	93.0	0.86	96.5	0.90
CNN + Transformer	97.0	0.88	97.0	0.91
**Proposed (Full)**	**97.0**	**0.882**	**98.5**	**0.92**

## 6 Conclusion

This work introduces a hybrid deepfake detection architecture that integrates CNN, bidirectional BiLSTM networks, and transformer encoders, which helps to jointly extract spatial and temporal features from video content. By preserving localized frame-level inequalities while modeling long-range temporal dependencies, the framework overcomes the limitations of single-modality approaches and demonstrates strong performance on benchmark datasets such as FaceForensics++ and DFDC, achieving high accuracy and robustness against compression, occlusion, and low-resolution scenarios. However, several significant challenges remain. First, the model is vulnerable to anti-motion, as subtle pixel-level manipulations produced by methods such as FGSM or PGD can fool recognition systems. To address this limitation, adversarial training, stochastic smoothing, defensive distillation, and Bayesian uncertainty estimation are required to improve resilience under adversarial. Second, real-time expansion poses computational challenges, as the quadratic self-attention complexity of the transformer becomes a bottleneck for long video sequences. Therefore, future work should investigate pruning, quantization, knowledge distillation, efficient linear-attention variants (e.g., Linformer, Performer, Longformer), and adaptive frame selection strategies to reduce latency without sacrificing accuracy. Another critical challenge concerns generalization to novel and unseen manipulation techniques. Deep learning detectors are often limited by dataset bias, where training on a limited subset of manipulations (e.g., face swaps or reenactments in FF++ or DFDC) limits robustness when exposed to new forgery strategies or unseen datasets. Future directions include domain adaptation frameworks to align feature distributions across datasets and self-supervised learning approaches that leverage large-scale unlabeled video corpora to learn transferable spatiotemporal representations. Cross-dataset validation protocols are also needed to ensure that detection performance generalizes to real-world situations beyond benchmark datasets. Furthermore, the interpretability of the framework needs to be improved. While the hybrid design is effective, its black-box nature limits transparency, which is crucial in forensic and legal contexts where trust and accountability are paramount. Incorporating interpretability techniques such as saliency maps, Grad-Cam, and attention visualization will make the decision process more transparent and increase user confidence. Finally, extending the framework towards multimodal deepfake detection by integrating audio-visual synchronization cues and physiological signals such as photoplethysmography (PPG) or breathing rhythms offers a promising path towards more comprehensive and reliable recognition systems.
